# Surgical protocol for precise and high-throughput viral injections in rhesus monkey brain

**DOI:** 10.1016/j.xpro.2026.104522

**Published:** 2026-04-28

**Authors:** Anne Claire Tangen, Peyton Harmon, Anya S. Plotnikova, Arya Mohanty, Alexander C. Cummins, Kenneth A. Pelkey, Katherine Cameron, Camille Rood, Kathryn Ann Guerriero, Chris J. McBain, Mark A.G. Eldridge, Bruno B. Averbeck, Reza Azadi

**Affiliations:** 1 Laboratory of Neuropsychology, National Institute of Mental Health, NIH, Bethesda, MD 20892, USA; 2 Neuroscience Institute, Carnegie Mellon University, Pittsburgh, PA 15213, USA; 3 Nash Family Department of Neuroscience and Friedman Brain Institute, Icahn School of Medicine at Mount Sinai, One Gustave L. Levy Place, New York, NY 10029, USA; 4 Lipschultz Center for Cognitive Neuroscience, Icahn School of Medicine at Mount Sinai, New York, NY 10029, USA; 5 School of Medicine, Wayne State University, Detroit, MI 48201, USA; 6 Eunice Kennedy Shriver National Institute of Child Health and Human Development, NIH, Bethesda, MD 20892, USA; 7 Section on Instrumentation, National Institute of Mental Health, NIH, Bethesda, MD 20892, USA; 8 Veterinary Medicine and Resources Branch, National Institute of Mental Health, NIH, Bethesda, MD 20892, USA; 9 Newcastle University, NE1 7RU Newcastle upon Tyne, UK

**Keywords:** Neuroscience, Cognitive Neuroscience, Behavior

## Abstract

Viral injections in nonhuman primates allow modification of neurons to investigate the organization and function of neural circuits, their developmental processes, and disease-related mechanisms. Here, we present a protocol for performing precise stereotaxic microinjections in the macaque brain. We describe steps for pre-surgical imaging, target planning, viral injections, and perioperative care. We then detail procedures for tissue extraction and slice electrophysiology experiments in multiple subcortical and cortical regions.

For complete details on the use and execution of this protocol, please refer to Furlanis et al.^1^

## Highlights

• Steps for MRI-guided viral-injection coordinate planning in macaques
• Instructions for building a high-throughput microinjection circuit
• Guidance on surgical procedures and perioperative care
• Procedure for brain extraction for *ex vivo* electrophysiology

## Abstract (editor highlights)

Publisher’s note: Undertaking any experimental protocol requires adherence to local institutional guidelines for laboratory safety and ethics.

## Graphical abstract

**Figure:**
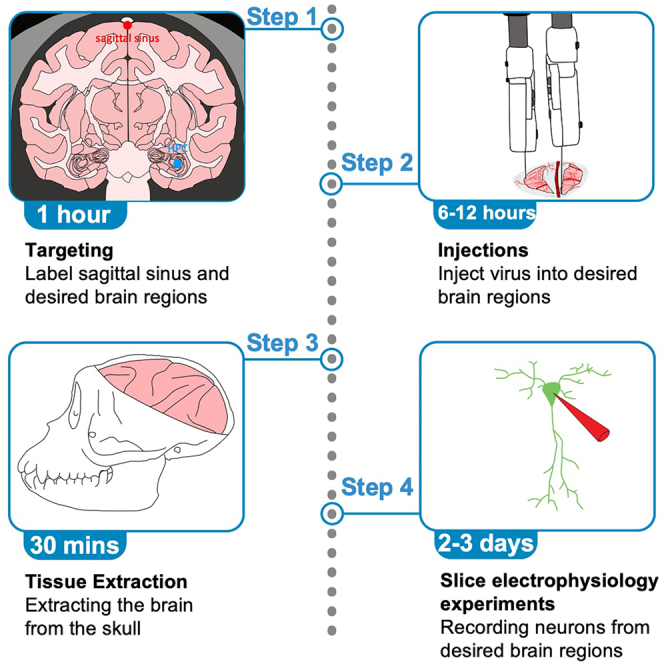

## Before you begin

Stereotaxic surgery is a common technique in systems neuroscience for performing precise microinjection in the nonhuman primate brain.^2^^,^^3^ Despite the wide use of stereotaxic procedures, detailed methods are rarely included in published studies. This protocol provides a step-by-step guide for performing high-precision stereotaxic viral injections in macaques. The procedures described here were developed and optimized for the experiments reported in previous publications.^1^^,^^4^^,^^5^^,^^6^^,^^7^^,^^8^ Using this approach, a wide range of viral vectors and payloads (e.g., GFP, RFP, ChR2) have been effectively delivered, including adeno-associated viruses (AAVs), particularly AAV-PHP.eB, an engineered AAV9 capsid variant, and lentiviral vectors. Although the core surgical framework is broadly applicable across viral vector types, injection parameters (e.g., volume, rate, and post-injection survival time) may require optimization depending on vector characteristics and experimental goals. In addition to viral injection, the same general framework can be adapted for other stereotaxic and surgical applications in nonhuman primates, including tracer injections, excitotoxic lesion studies, and chronic cranial implantations. Beyond the injection procedure, this protocol describes how to extract the brain for ex vivo electrophysiological studies, which differs from commonly used post-euthanasia extraction used for anatomical or histological analyses. We also introduce a custom-designed injection circuit that enables simultaneous multiple injections, improving reliability and substantially reducing surgical duration. In developing this protocol, we incorporated and expanded upon established methods in the literature, such as the use of tooth markers,^9^ and sagittal sinus alignment.^10^ Finally, we further optimized the previously published surgical protocols^11^^,^^12^^,^^13^ and injection-circuit designs.^14^ All procedures were conducted in accordance with the guidelines approved by the National Institute of Mental Health Animal Care and Use Committee.

### Innovation

We present a new injection circuit that can be used across a range of surgical procedures requiring microinjections, including injections delivered through open cranial chambers. In addition, we provide a detailed protocol for general stereotaxic surgeries, addressing several aspects that are often underreported.

### Institutional permissions

All procedures were conducted in accordance with the guidelines approved by the National Institute of Mental Health Animal Care and Use Committee. Researchers intending to replicate these procedures must obtain prior approval from their own regulatory bodies.

## Key resources table

**Table:** 

REAGENT or RESOURCE	SOURCE	IDENTIFIER
**Bacterial and virus strains**		

Experimental viral vector(s)	N/A	N/A

**Chemicals, peptides, and recombinant proteins**		

Glucose	MilliporeSigma	Cat#G7528
Sodium Bicarbonate	MilliporeSigma	Cat#S5761
Ascorbic Acid	MilliporeSigma	Cat#11140
Sodium chloride	MilliporeSigma	Cat#S7653
Monosodium Phosphate	MilliporeSigma	Cat#S3139
Potassium Chloride	MilliporeSigma	Cat#P9333
Calcium Chloride	MilliporeSigma	Cat#C5080
Magnesium Chloride	MilliporeSigma	Cat#M9272
Water	MilliporeSigma	Cat#320072

**Experimental models: Organisms/strains**		

Rhesus macaque, *macaca mulatta*	National Institute of Mental Health	N/A

**Software and algorithms**		

Mango	Multi-image Analysis GUI	https://mangoviewer.com/mango.html
Microsoft Excel	Microsoft	RRID: SCR_016137

**Other**		

Large animal stereotax	Jerry-Rig USA	Cat#JR-MRIC
Accura ClearVue	3D Systems, Inc.	N/A
MED Digital ABS	Stratasys	N/A
Passivated 18-8 Stainless Steel Phillips Pan Head Screw #2**-**56 Thread Size, 5/16″ Long	McMaster-Carr	Cat#91772A078
Passivated 18-8 Stainless Steel Phillips Flat Head Screw #4-40 Thread, 5/16″ Long	McMaster-Carr	Cat#91771A107
18-8 Stainless Steel Socket Head Screw, #4-40 Thread Size, 3/8″ Long	McMaster-Carr	Cat#92196A108
Corrosion-Resistant 18**-**8 Stainless Steel Hex Nuts, #2**-**56 Thread Size	McMaster-Carr	Cat#91841A003
Corrosion-Resistant 18**-**8 Stainless Steel Hex Nuts, #4**-**40 Thread Size	McMaster-Carr	Cat#91841A005
18**-**8 Stainless Steel Square Nut, #4**-**40 Thread Size	McMaster-Carr	Cat#94785A511
30-gauge precut, 45 degrees, lancet-style sharpening needles	Vita Needle	N/A
23-gauge 304 Stainless Steel Tubing, Miniature, 0.025″ OD, 0.004″ Wall Thickness	McMaster-Carr	Cat#8987K64
Uncoated High-Speed Steel Drill Bit, 0.65mm Size, 32mm Overall Length	McMaster-Carr	Cat#2951A44
50 cm PEEK tubing 1/16″ OD, 0.007″ ID	IDEX Health & Science	Cat#1536 L
Plastic Tubing Cutter	IDEX Health & Science	Cat#A-327
CapTite Microfluidic Interconnect Elbow piece	LabSmith	Cat#T116-205
Microfluidic CapTite One-Piece Fitting ferrule	LabSmith	Cat#T116-100
CapTite Two-Piece Adapter	LabSmith	Cat#T116-A360
IDEX Fingertight One-Piece Fitting, Standard Knurl, Natural PEEK, 1/16″ OD Tubing, 10**-**32 Coned	IDEX Health & Science	Cat#F-120X
IDEX Threaded Adapter, PEEK, 0.020″ ID, 10**-**32 Coned (F) to 1/4**-**28 Flat Bottom (F)	IDEX Health & Science	Cat#P-627-01
100 μL, Model 1710 CX Syringe, 1/4**-**28 Threads, Plunger Stop	Hamilton	Cat#81062
Electrode Manipulators Models 1460, 1460**-**61	Kopf	Cat#1460-61
Medline Sterile 100% Cotton Woven Gauze Sponges (4x4 sterilized gauzes)	Medline	Cat#NON21420
Standard Back Table Cover	Halyard	Cat#42217
Disposable Flexible Light Handle Cover	Medline	Cat#DYNJLHS1H
Skin Prep Solution 3 M™ DuraPrep™ 6 mL	3 M	Cat#3M-8635
Utility Marker with Ruler	Medline	Cat#DYNJSM06
Disposable Utility Drapes	Medline	Cat#DYNJP2405
Disposable Craniotomy Drape	Medline	Cat#DYNJP10001H
3 M™ Ioban™ 2 Antimicrobial Incise Drape	Solventum Corporation	Cat#6650EZSB
Disposable Suction Coagulator Handswitch	Ambler Surgical	Cat#SCH0 8
Disposable Bulb Syringe	Cardinal Health	Cat#67000
EZ Drape Sterile Hose & Cable Covers	AD Surgical	Cat#A400-1000
Kendall Disposable 16oz Solution Bowl, Covidien	Cardinal Health	Cat#61000
Kendall Disposable 32 oz Solution Bowl, Covidien	Cardinal Health	Cat#61200
Electrosurgical Pencil Argent™ 10 Foot Cord Blade Tip	McKesson	Cat#22-ESP1
Sterile Scalpel Blades- #11	Roboz Surgical Instrument Company	Cat#RS-9801-11
Sterile Scalpel Blades- #10	Roboz Surgical Instrument Company	Cat#RS-9801-10
Sterile Scalpel Blades- #15	Roboz Surgical Instrument Company	Cat#RS-9801-15
Scalpel Handle, #3; Solid; 4″ Length	Roboz Surgical Instrument Company	Cat#RS-9843
Scalpel Handle, #7; Solid; 6.25″ Length	Roboz Surgical Instrument Company	Cat#RS-9847
Tissue Forceps; 1X2 Teeth; 5.5″ Length; 2 mm Tip Width	Roboz Surgical Instrument Company	Cat#RS-8164
Freer Periosteal Elevator, Sharp/Blunt, 7.5″	Black & Black Surgical, Inc.	Cat#B 63876
Freer Elevator, Double Ended, Sharp/Blunt, 7″	Roboz Surgical Instrument Company	Cat#RS-8820
Halsted Mosquito Forceps; Curved; 5″	Roboz Surgical Instrument Company	Cat#RS-7111
Hartman Hemostatic Mosquito Forceps- 3.5″	Ambler Surgical	Cat#30-851
Mayo Scissors, Straight, 5.5″	Roboz Surgical Instrument Company	Cat#RS-6870
Small Muscle Retractor, Spratt Curette, Size 5/0	Roboz Surgical Instrument Company	Cat#RS-9040
Large Muscle Retractor, Spratt Curette, Size 2/0	Roboz Surgical Instrument Company	Cat#RS-9052
High Speed Surgical Drill Set 120 VAC	BASi	Cat#MF-5360
Circular Saw Cutting Wheels	Pfingst & Company	Cat#H041047 (S), H041061 (M), H041073 (L)
Drill Bits	Roboz Surgical Instrument Company	Cat#RS-6280C-2 (#2), RS-6280C-5 (#5), RS-6280C-8 (#8)
Stainless Steel Surgical Spoons	Jerry-Rig USA	Cat#JR-SSSS
RUSKIN Rongeur; Double Action, Curved; 3mm Jaw Width; 6″ Length	Roboz Surgical Instrument Company	Cat#RS-8431
BoneSeal Absorbable Bone Hemostat	Terumo Cardiovascular	Cat#9550054
Bishop-Harmon Tissue Forceps- 3 3/8″, straight shafts, delicate, 0.5mm 1x2 teeth, flat 3-hole handle, titanium	Ambler Surgical	Cat#9567 T
Micro Dissecting Scissors, Straight, 3.5″	Roboz Surgical Instrument Company	Cat#RS-5907
Castroviejo Needle Holder, Curved, 5.5″	Ambler Surgical	Cat#50-171
Frazier Micro Dissecting Hook; Sharp; 5″ Length; 3mm Loop	Roboz Surgical Instrument Company	Cat#RS-6180
Suction Tube 18 Gauge Bent + Controller	Neozoline	Cat#NZ4251Bent
Artificial Dura Tecoflex EG-93 A Thickness 0.005″	Lubrizol	Cat#EG-93 A
Codman Surgical Patties, 13 x 76 mm	Integra	Cat#801407
Backhaus Towel Clamp; 3.5″	Roboz Surgical Instrument Company	Cat#RS-7780
Littauer Stitch Scissors; 5.5″	Roboz Surgical Instrument Company	Cat#RS-7076
Weitlaner Retractor; 3 X 4 Blunt Prongs, 8″	Roboz Surgical Instrument Company	Cat#RS-8616
Mayo-Hegar Needle Holder With Carbide Jaws; 6″	Roboz Surgical Instrument Company	Cat#RS-7922
Harvard Apparatus Pump Controller (HAPC)	Harvard Apparatus	Cat#70-4404
Nanomite Injector, Single Syringe, Black	Harvard Apparatus	Cat#70-3602
Suture 2**-**0 PDS II Violet	Ethicon	Cat#Z997G
ETHILON® Nylon Suture 3**-**0	Ethicon	Cat#1663G
COATED VICRYL® (polyglactin 910) Suture 3-0	Ethicon	Cat#J316H
PROLENE Polypropylene Suture 4**-**0	Ethicon	Cat#D7143
ETHILON® Nylon Suture 5**-**0	Ethicon	Cat#1855G
COATED VICRYL® (polyglactin 910) Suture 5**-**0	Ethicon	Cat#J493G
PRANG™	Bio-Serv	Cat#F2351

## Materials and equipment

Sucrose-substituted artificial cerebrospinal fluid (ssACSF); saturated with carbogen (95% O2, 5% CO2), with osmolarity 310**–**320 Osm.

**Table:** 

Reagent	Final concentration (% w/v)	Amount
Sucrose	3.08%	90 mM
NaCl	0.47%	80 mM
NaH_2_PO_4_	0.015%	1.25 mM
KCl	0.026%	3.5 mM
CaCl_2_	0.007%	0.5 mM
MgCl_2_	0.092%	4.5 mM
NaHCO_3_	0.20%	24 mM
Glucose	0.18%	10 mM
Water (H_2_O)	N/A	1 L

Store at 4°C for up to 3**–**5 days. Bubble with carbogen (95% O2, 5% CO2) immediately prior to use and cool for 0°C**–**4°C.

## Step-by-step method details

### Building the injection setup

**Timing: 30 min**

**Figure 1:**
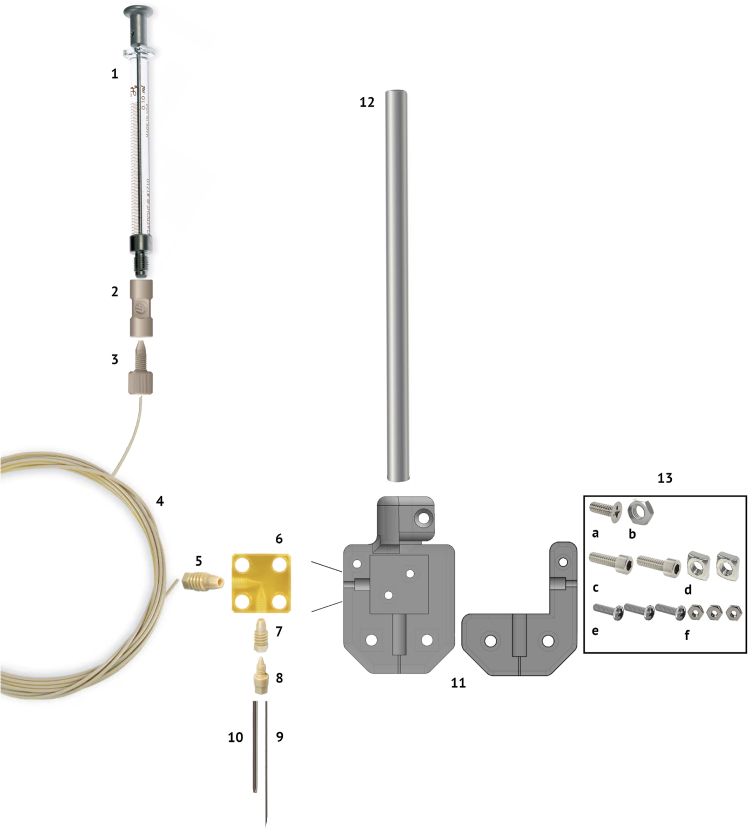
Injection circuit components Hamilton 100 μL syringe (1), Idex Threaded Adapter (2), Idex Fingertight One-Piece Fitting (3), 50 cm PEEK tubing 1/16” (4), Microfluidic CapTite One-Piece Fitting ferrule (5), CapTite Microfluidic Interconnect Elbow piece (6), CapTite Two-Piece Adapter (7, 8), Hamilton 30 gauge precut, 45 degrees, lancet-style sharpening needle (9), 23 gauge 304 Stainless Steel Tubing guide tube (10), 3D-printed injection circuit holder (11), metal bar which connects to micromanipulator (12), Phillips Flat Head Screw (4**-**40 Thread, 5/16″ Long; 13a), Hex Nuts (4**-**40 Thread Size; 13b), Socket Head Screws (4-40 Thread Size, 3/8″ Long; 13c), Square Nuts (4-40 Thread Size; 13d), Phillips Rounded Head Screws (2**-**56 Thread Size, 5/16″ Long; 13e), Hex Nuts (2**-**56 Thread Size; 13f).

Figure 1 illustrates the components of the injection setup.

This section describes the assembly of a custom injection system for reliable delivery of solutions into the brain, enabling precise and simultaneous injections in parallel across multiple sites. It includes fabrication of the 3D-printed holder, construction of the injection circuit and loading connectors, and validation of the system to ensure secure connections, absence of leaks, and proper fluid flow prior to surgical use.

1. Building the 3D-printed injection circuit holder:
  a. Use the provided STL file (Methods S1) to 3D-print the circuit holder in Accura ClearVue from 3D Systems (only Ethylene oxide (EtO) gas compatible).
  b. Attach the bar to the 3D-printed holder by tightening the #4-40 x 5/16″ Phillips Flat Head Screw into the #4-40 hex nut to secure the 3D-printed holder to the metal bar.

**CRITICAL:** Verify that the connection between the 3D-printed holder and metal bar is secure and does not rotate or move during use.

2. Building the injection circuits:
  a. To prepare the injector needle, sharpen the tip of a hypodermic tube to a 30° bevel. Alternatively, use a pre-sharpened hypodermic needle.
  b. Cut a 30-gauge hypodermic needle to a length of 70 mm.
  c. Cut a 23-gauge needle to a length of 50 mm.
  d. Use a CapTite Two-Piece Adapter to connect the needle to CapTite Interconnect Elbow.
  e. Place the 23-gauge guide tube around the 30-gauge injector needle to protect it from bending during penetration of the brain.
  f. Use a Plastic Tubing Cutter to cut the PEEK tubing to a length of approximately 50 cm, ensuring a flat and burr-free cut.
  g. Connect the PEEK tubing to the CapTite Interconnect Elbow using a CapTite One-Piece Fitting (Ferrule).
  h. Secure the CapTite Interconnect Elbow into the 3D-printed connector holder using Phillips Rounded Head Screws (2-56 Thread Size, 5/16″ Long) and Hex Nuts (2-56 Thread Size).
  i. Attach the top piece of the 3D print using Socket Head Screws (4-40 Thread Size, 3/8″ Long), hex nuts (4-40 Thread Size), Phillips Rounded Head Screw (2-56 Thread Size, 5/16″ Long), and hex nut (2-56 Thread Size).
  j. Attach an IDEX Fingertight One-Piece Fitting to the other end of the PEEK tubing.
  k. Connect this fitting to an IDEX Threaded Adapter, PEEK, 0.020″ ID, 10-32 Coned (F) to 1/4-28 Flat Bottom (F). Slide the PEEK tubing out at least 5 mm before screwing it.
3. Building the loading connectors:
  a. Cut a 23-gauge hypodermic tubing to a length of 65 mm.
  b. Cut a piece of PEEK tubing to approximately 5mm.
  c. Drill through the PEEK tubing using an approximately 0.6 mm drill bit to allow insertion of the 23-gauge needle.
  d. Insert the needle into the PEEK tubing, then place the PEEK-covered end into an IDEX Fingertight One-Piece Fitting, with the narrow side facing outward to allow secure insertion into the IDEX adapters.
  e. Screw this assembly into the coned side of the IDEX Threaded Adapter (PEEK, 0.020″ ID, 10-32 Coned (F) to 1/4-28 Flat Bottom (F)).
  f. Attach a syringe to the flat-bottom side of the adapter.
4. Testing the injector circuit:
  a. Verify all connections by gently pulling on the PEEK and hypodermic tubes to ensure they are secure.
  b. Using the loading connector, fill a syringe with distilled water.
  c. Inject the distilled water through the circuit to confirm there are no clogs, and check all connectors for leaks.

**CRITICAL:** PEEK connectors should be tightened only finger-tight; overtightening can cause cracks or deformation. Ensure that the needle tip remains straight and sharp through assembly.

***Note:*** See troubleshooting 1 to remedy a clogged injection circuit.

### Pre-operative scanning

**Timing: 3 h**

This section describes positioning the animal in an MR-compatible stereotaxic frame and acquiring pre-operative imaging for accurate targeting of brain structures. Proper head fixation and alignment are critical to ensure reliable stereotaxic coordinates and proper registration between imaging and surgical procedures.

5. Framing the animal in an MR-compatible stereotaxic device.
  a. With one ear bar firmly secured in place as far back against the stereotaxic arm as possible, lift the monkey’s head and feel for the zygomatic arch.
  b. Place the ear bar into the monkey’s ear canal.
    ***Note:*** To ensure the ear bar is correctly placed 127 into the ear canal, you should be able to feel the end of the zygomatic arch against 128 and perpendicular to the ear bar.
  c. While holding the head against the ear bar in the ear canal, place the other ear bar in the contralateral ear canal using the same method by feeling for the zygomatic arch.
  d. Place that ear bar into the respective cavity and secure it in the frame.
  e. With the help of another person, align and center the ear bars by loosening both ear bar-locking screws.
    **CRITICAL:** Maintain steady inward pressure on both ear bars at all times to prevent them from slipping out of the ear canals.
  f. Adjust the ear bars until the monkey’s head is centered in the frame.
    **CRITICAL:** Confirm that the numbers visible on the ear bars match, indicating symmetric ear bar placement and proper midline alignment of the head.
  g. Place the orbit holder and mouthpiece by loosening both screws. Place the mouth-piece at the roof of the monkey’s mouth without placing the piece under the tongue. Simultaneously, hook the orbit holders on the lower orbits of the monkey.
  h. While pulling up on the mouthpiece as much as possible, secure both screws, starting with the anterior-posterior (AP) adjustment screw.
  i. Record the ear bar and the orbit holder positions.
  j. Finally, place a tooth marker on the tip of one of the monkey’s canines or another notable tooth in the upper jaw.^10^
  k. After positioning and securing the animal in the stereotaxic frame, transfer the animal to the scanner and acquire imaging data.
    **CRITICAL:** Ensure that both ear bars are seated correctly in the ear canals. An improperly positioned ear bar will lead to inaccurate head alignment and unreliable stereotaxic measurements. If there is any doubt about placement, reframe the animal and recheck the tooth-marker measurements.

### Targeting

**Timing: 1 h**

This section describes approaches for identifying and targeting cortical and subcortical brain regions for injection. While dorsal cortical areas can be localized visually during surgery, deeper structures require stereotaxic targeting based on pre-operative MRI. Accurate determination of coordinates relative to anatomical landmarks ensures precise delivery of viral constructs to the intended brain regions.

6. Targeting dorsal cortical areas can be performed visually by exposing the cortical surface during surgery. Using approximately 10 μL of viral solution is typically sufficient to transfect a circular region of cortex about 2 mm in diameter.

***Note:*** This volume may be adjusted depending on the targeted cortical area, viral construct, and specific experimental goals.

**Figure 2:**
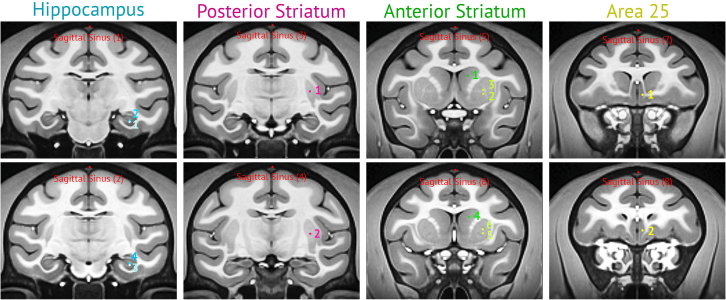
Stereotaxic target placement

7. Targeting subcortical structures using stereotaxic localization. The following steps describe how to determine each injection site:
  a. Identify target structures (e.g., hippocampus, posterior striatum, anterior striatum, area 25) on the pre-operative MRI.
  b. See Figure 2, Table 1, and Supplementary file 2 for predefined injection coordinates, volumes, and rates (generated using Mango software on the NMT v2 template).

**Table 1:** Injection parameters

Injection #	Volume (μL)	Rate (μL/min)	Injection duration (min)	Post-injection hold time (min)
Hippocampus				
1	10	1	10	1
2	10	1	10	10
3	10	1	10	1
4	10	1	10	10
Posterior Striatum				
1	20	1	20	10
2	20	1	20	10
Anterior Striatum				
1	10	1	10	10
2	10	1	10	1
3	10	1	10	10
4	10	1	10	10
5	10	1	10	1
6	20	1	20	10
Area 25				
1	20	1	20	10
2	20	1	20	10

Note that these injection parameters are not intended to uniformly cover the entire volume of the targeted structures; however, they reliably produce sufficient levels of transfection for the studies cited in this paper.
  c. Using sagittal slices from the pre-operative MRI, identify the Y-coordinate (rostro-caudal axis) corresponding to the tips of the ear bars.
  d. For each target, determine the X (medio-lateral, ML), Y (rostro-caudal/anterior-posterior, AP), and Z (dorso-ventral, DV) coordinates of the injection site, as well as the X, Y, and Z coordinates of the sagittal sinus on the same coronal slice.
  e. During surgery, calculate the Y coordinate relative to the ear bar position (“ear bar zero”), and determine the X and Z coordinates using the sagittal sinus as a reference.
  f. Use the provided Excel sheet (Methods S2) to facilitate the calculation of stereotaxic coordinates for target sites during surgery.

***Note:*** Here we provide details of targeting subcortical structures, such as hippocampus, posterior striatum, anterior striatum and area 25. The details of targeting parameters (i.e., injection coordinates, volumes, and rates) are provided in Figure 2 and Supplementary file 2, which was generated using Mango software on a macaque template: NMT v2.^15^^,^^16^Table 1 summarizes the injection parameters for each target.

### Sterilizing surgical instruments

**Timing: 15 h**

This section describes sterilization of all surgical instruments and components required for the procedure. Proper sterilization is essential to maintain aseptic conditions, prevent contamination, and ensure reliable surgical and injection performance.

8. Sterilize the following items:
  a. Surgical instruments.
  b. Injection circuits, loading connectors, and spare components.
  c. Microinjection syringes.
  d. Allen wrenches for adjusting the micromanipulator components if necessary.
  e. Microinjection pumps.
  f. Micro-manipulators.
  g. Ear bars.

**CRITICAL:** If you sterilize the injection circuits with Ethylene Oxide (EtO), thoroughly flush the circuits with alcohol solution followed by multiple rinses with distilled water, and then flush air to ensure the internal lumen is clean and completely dry. This step is necessary to allow reliable penetration of EtO gas into the circuit.

### Loading virus into syringes

**Timing: 1 h**

This section describes loading viral solution into the injection system while minimizing dead volume and preventing dilution or contamination.

9. Pre-filling the injection circuit to minimize dead volume (optional); If needed, the injection circuit can be pre-filled with sterile mineral oil to minimize dead volume, as described below.
  a. Under sterile conditions, attach the loading connector to the syringe.
  b. Place the hypodermic tubing into sterile mineral oil and draw back the plunger to load mineral oil into the syringe.
  c. Push and pull the plunger several times to eliminate air bubbles completely.
  d. Draw up approximately 20 μL of sterile mineral oil.

***Note:*** The dead volume of the injection circuit is approximately 25 μL. This volume can be reduced, if necessary, by shortening the PEEK tubing and decreasing its internal diameter, as well as the needle length. However, a significant portion of the dead volume results from the syringe connector (approximately 10 μL).

10. Without removing the loading connector, wipe the needle tip to remove residual oil before inserting it into the virus vial. Withdraw the required volume of viral solution.
11. Remove the loading connector and draw back the plunger to create a 10 μL air gap, ensuring no liquid remains in the needle or connector.

**CRITICAL:** It is important to completely get rid of the air bubble gap when initially loading the mineral oil. Because of the dead volume of the loading connector, after you have loaded 5 μL into the syringe, remove the needle tip from the mineral oil to then proceed with loading virus.

### Supportive medications

**Timing: 30 min**

This section describes the administration of supportive medications to reduce inflammation, prevent infection, and manage pain during the perioperative period. This protocol is used to promote recovery, minimize complications, and ensure animal welfare before and after surgery.

12. Administer pre- and post-operative medications, including corticosteroid, antibiotic, and analgesic agents, as described below.
  a. Corticosteroids:
    i. Administer dexamethasone 0.5 mg/kg IM the evening prior to surgery.
    ii. From the day of surgery, continue administration at 0.5 mg/kg three times daily (TID) for 3 days, followed by 0.5 mg/kg twice daily (BID) for 2 days, then 0.5 mg/kg once daily (SID) for 1 day, and finally 0.25 mg/kg once daily (SID) for 1 day.
      ***Note:*** This tapering schedule helps minimize post-operative neuroinflammation and minimize edema.
  b. Antibiotics:
    i. Administer cefazolin 25 mg/kg IM the evening prior to surgery.
    ii. Beginning on the day of surgery, continue cefazolin 25 mg/kg IM twice daily (BID) for 3 days, followed by cephalexin 25 mg/kg PO twice daily (BID) for 11 days.
  c. NSAIDs:
    i. Administer meloxicam 0.2 mg/kg IM once daily (SID) for 3 days starting on the day of surgery, followed by meloxicam 0.2 mg/kg PO once daily for an additional 3 days.
      ***Note:*** Injectable cefazolin is used initially to ensure reliable drug delivery during early post-operative hyporexia; once appetite normalizes, treatment is switched to oral cephalexin to minimize restraint associated with injections.

### Zeroing the stereotaxic frame using the ear bar

**Timing: 30 min**

This section describes establishing a stereotaxic reference (“zero”) using the ear bars to align the injection system with the coordinate space defined during MRI.

**Figure 3:**
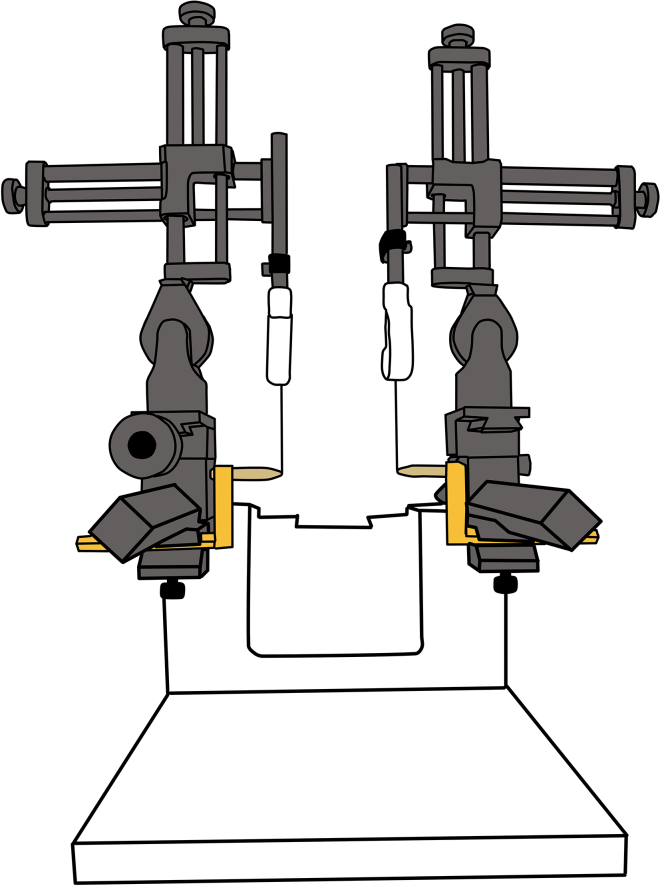
Zeroing the stereotaxic frame

Figure 3 illustrates the zeroing procedure.

13. Under sterile conditions, place the sterilized ear bars in the stereotaxic frame and adjust them to the same distance used during MRI scanning.

**CRITICAL:** Ensure to maintain sterility of the ear bar tip, as the injection needle may accidentally touch them.

14. Mount the sterilized micromanipulators onto the stereotaxic arms.
15. Attach the injection circuit to the micromanipulator.
16. Using the micromanipulator, move the needle tip until it is precisely aligned with the center or the tip of the ear bar and record the corresponding coordinates.

### Induction of anesthesia

**Timing: 30 min**

This section describes induction of anesthesia and preparation of the animal for surgery. Proper anesthesia and supportive care are essential to ensure stability, safety, and optimal surgical conditions.

17. For initial sedation, anesthetize the animal with ketamine 5**–**15 mg/kg and midazolam 0.05–0.2 mg/kg IM, then transport the animal to the surgical preparation area.

***Note:*** Alternative sedation regimens may be used as appropriate in consultation with the institute veterinarian.

18. Place an intravenous catheter in a peripheral vein, such as the saphenous or cephalic vein, and proceed with endotracheal intubation.
19. Collect perioperative blood samples for complete blood count (CBC) and serum biochemistry analysis.
20. Shave the head and neck using clippers, optionally followed by application of a depilatory cream, then perform an initial rough scrub to remove all hair and debris.

***Note:*** Tape or a lint roller can be used to remove any remaining hair or debris.

21. Transport the animal to the operating room once prepared.
22. Monitor physiological parameters throughout the procedure, including heart rate and electrocardiogram, end-tidal CO2, respiratory rate, blood pressure, body temperature, and minimum alveolar concentration (MAC).

***Note:*** If needed, connect the animal to a ventilator to maintain stable respiration.

23. Maintain intravenous catheter patency by infusing lactated Ringer’s solution at 3**–**5 ml/kg/hour.
24. Administer mannitol 2 g/kg of a 20% solution IV over 20 min, two to three times during surgery, depending on the degree of brain swelling observed.

### Initial prepping and framing

**Timing: 45 min**

This section describes preparation of the surgical site, including skin disinfection, sterile draping, and setup of surgical instruments and equipment. Proper preparation and maintenance of sterility are critical to minimize contamination risk and ensure optimal surgical conditions.

25. Prepping the scalp:
  a. In a sterile manner, place 10 to 20 4x4 sterilized gauzes in two separate sterile bowls.
  b. Fill one bowl with 70% ethanol and the other with a surgical scrub solution (e.g., Povidone-Iodine scrub).
  c. In a sterile manner, protect the animal’s eyes by placing a dry, sterile gauze over the eyes.
  d. Using the dominant hand, scrub the surgical site in a concentric circular motion, starting from the center of the skull and moving outward, with a gauze soaked in the scrub solution.
    **CRITICAL:** Avoid direct contact between your gloved hand and the animal skin.
  e. Use the alcohol-soaked gauze (from the second bowl) to remove the scrub solution from the skin.
  f. Use sterile forceps, or your non-dominant hand, to access the bowls.
  g. Also, use your non-dominant hand to ensure the gauze protecting the eyes remains in place.
  h. Repeat the scrub-rinse cycle at least three times, or until all visible debris and remaining trimmed hairs are removed from the skin.
  i. Use the same method to disinfect the stereotaxic frame arms.
    **CRITICAL:** Do not use the same gauze pads that were used on the skin for cleaning the equipment to prevent cross-contamination.
  j. Finally, apply a sterile antiseptic solution (e.g., DuraPrep) to the prepared skin, following the manufacturer’s recommended contact time and usage instructions.
  k. Allow the solution to dry fully before draping.
    **CRITICAL:** Always protect the eyes throughout the prepping procedure, as the scrub solution can damage the cornea. Before draping, make sure no loose hair or debris remains on the skin. Proper skin preparation is also essential for adhesive drapes to adhere securely and remain in place throughout the surgery.
26. Scrub your hands and put on your sterile gown and gloves.
27. Draping of the surgical field:
  a. In a sterile manner, place the disposable flexible light handle covers on the lights and move them to focus on the surgical site.
  b. Using a sterile skin marker, mark the sagittal midline, then define the incision boundaries: rostrally at the level of the brow ridge and caudally extending at least 20 mm posterior to the occipital ridge.
    ***Note:*** A longer incision facilitates lateral retraction of the skin, improving the exposure of the skull and temporalis muscles.
  c. For the initial draping layer, use three disposable utility drapes with sterile tapes.
  d. Place two drapes parallel to the sagittal midline, approximately 20 mm away to allow for skin stretching after incision.
  e. Place the third drape just anterior to the rostral incision mark.
    ***Note:*** If needed, secure drapes in place using towel clamps, grabbing the towel corners together with the monkey’s skin.
  f. Place the craniotomy drape by aligning the leading edge of the adhesive area with the anterior incision mark.
  g. Press down firmly to ensure full adhesion and eliminate any air bubbles.
    ***Note:*** Alternatively, an antimicrobial incision drape (e.g.3M™Ioban™) can be used to cover the surgical site, following the same protocol.
  h. Next, use general surgical drapes or table covers with a circular opening to cover the entire surgical field.
    ***Note:*** If needed, secure this outer layer with towel clamps, grabbing the towel corners together with the monkey’s skin.
  i. Alternatively, place a craniotomy drape by aligning the leading edge of the adhesive area with the anterior incision mark. Press down firmly to ensure full adhesion and eliminate any air bubbles.
    **CRITICAL:** Avoid touching the animal’s skin, the stereotaxic frame, or any other non-sterile surface while draping. If your gloves or gown become contaminated at any point, you will need to rescrub, put on new sterile gloves and gown, and re-drape the animal before proceeding.
28. Surgical site set-up:
  a. Connect the suction tip to the suction hose and attach the hose to the suction collection canister.
  b. Plug in the disposable cautery pen to the power source and confirm that it is functioning properly.
  c. Place two surgical bowls and a disposable bulb syringe filled with sterile saline on the Mayo stand.
  d. Arrange all instruments necessary for the opening phase on the Mayo stand for easy access.

### Surgical procedure

**Timing: 5–12 h**

This section describes the complete surgical procedure, including incisions, craniotomy, targeting, viral delivery, and closure. It provides a detailed, step-by-step guide to ensure accurate targeting, minimize tissue damage, and promote successful recovery.

29. Opening skin:
  a. Using a #10 surgical blade, make a single sagittal incision along the midline, carefully following the line that was marked during preparation (Figure 4).

**Figure 4:**
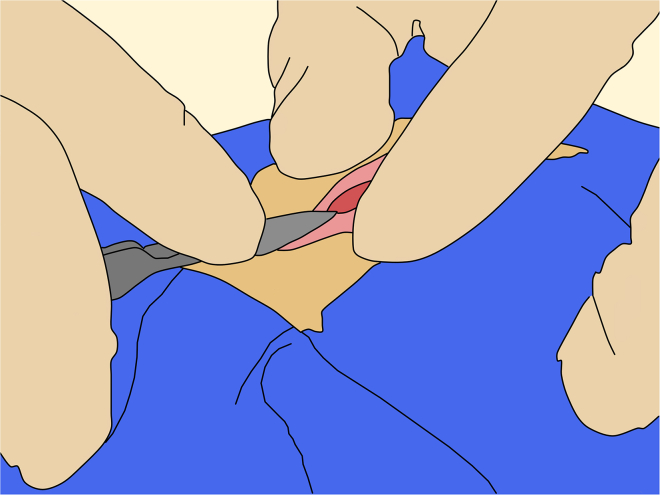
Skin incision
  b. Using the thumb and index finger of the other hand, maintain gentle pressure on the sides of the wound to keep it slightly open.
    **CRITICAL:** To preserve the galea, inspect the incision after the first few millimeters and adjust the depth as needed. If bleeding occurs, apply pressure with sterile gauze for hemostasis.
  c. Maintain visualizing the incision depth throughout and aim to complete the cut in a single continuous motion without lifting the blade from the skin (Figure 4).
  d. Do not separate the skin from the galea.
    ***Note:*** Doing so will create dead space that increases the risk of post-operative oedema.
30. Opening galea:
  a. Press the sharp end of the periosteal elevator into the sagittal midline to cut through the galea and expose the underlying bone for a few millimeters.
  b. Use the elevator to separate the galea from the skull along the midline, working through the incision.
  c. Once elevated, cut the galea along the periosteal elevator using Mayo scissors (Figure 5).

**Figure 5:**
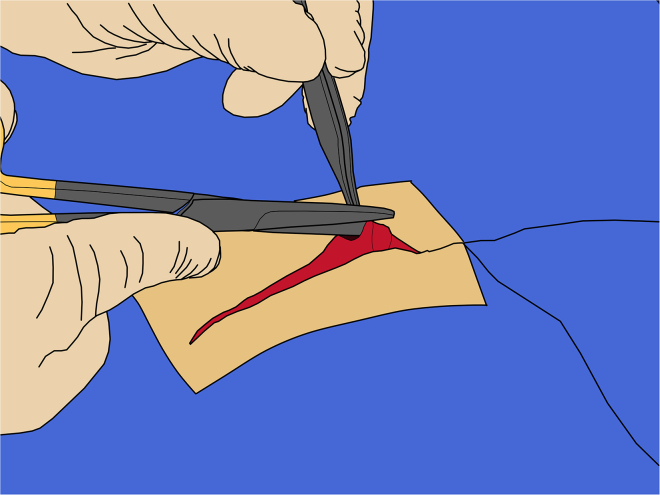
Incision of the galea
  d. Rather than using a cutting motion, open the scissors slightly, just a few millimeters, and slide them forward to create a smooth incision, minimizing localized tissue trauma while producing a smooth, continuous incision rather than a jagged cut caused by repeated opening and closing of the scissors.

***Note:*** Alternatively, make an incision with the #15 blade, following the line of the skin incision.

31. Retracting galea:
  a. Separate the galea from the deep temporalis fascia using a periosteal elevator, or Mayo scissors by inserting the closed blades and gently spreading them apart.
  b. Apply traction on the galea to facilitate dissection.

**Figure 6:**
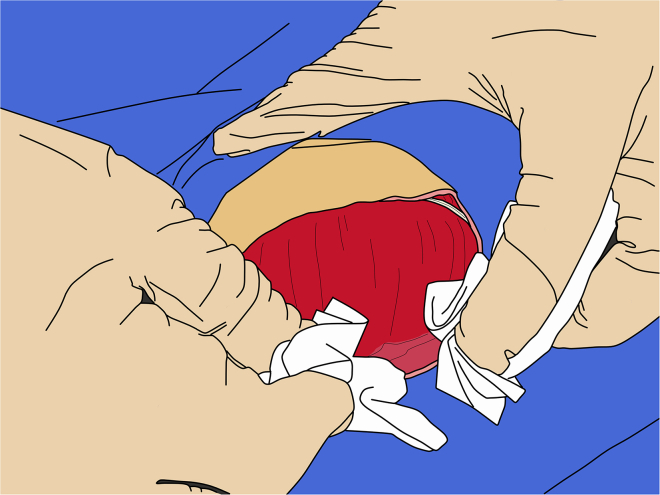
Retraction of the galea

***Note:*** Following initial separation, dry gauze can be used to separate the galea by gently pushing it laterally (Figure 6).

**Figure 7:**
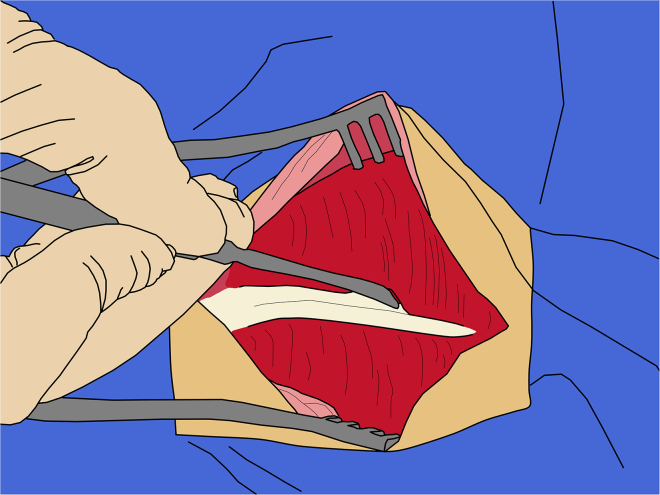
Retraction of the temporalis muscles
  c. Then, using muscle curettes gradually detach the body of the temporalis muscles from the skull.

***Note:*** Alternatively, the periosteal elevator can be used with controlled horizontal movements.

**Figure 8:**
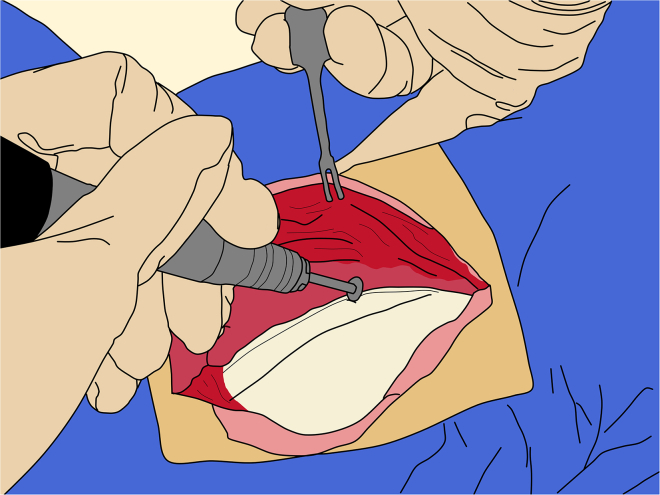
Craniotomy
    Injury to the sagittal sinus or its major branches can result in severe hemorrhage and may be life-threatening for the animal. If there is any uncertainty, use a bore drill bit for the central skull region to reduce the risk of dural or vascular damage.
  c. Once the margins are complete, use two periosteal elevators to gradually lift and detach the bone flap (Figure 9).
    **CRITICAL:** It is recommended to begin elevating from the caudal side, carefully monitoring for dural adhesions commonly found over the sagittal sinus, but elevation can occur anywhere.

32. Retracting temporalis muscles:
  a. Using the sharp edge of a periosteal elevator, detach the dorsal portion of the temporalis tendons from the skull, keeping the elevator oriented sagittally to minimize muscle trauma.
  b. After the initial detachment, gradually elevate the tendon to fully separate it from the bone (Figure 7).

33. Craniotomy:
  ***Note:*** Drilling and removing a large bone flap enables high-throughput and multiple injections, as well as visualization and measurement of sagittal sinus, improving targeting accuracy. Alternatively, this approach is not strictly required; burr holes can be used, as described in McBride et al.^11^
  The craniotomy margins should extend approximately 10 mm beyond the planned injection sites.
  a. Use a surgical ruler to measure the anterior-posterior boundaries relative to the ear bar level, and medio-lateral boundaries from the sagittal midline.
    ***Note:*** For bilateral injections, the craniotomy can be made symmetric; otherwise, the craniotomy should cross the sagittal sinus by approximately 10 mm.
  b. Using an ultrasonic piezoelectric bone cutter or a circular cutting wheel (Figure 8), begin drilling the craniotomy.
    **CRITICAL:** It is recommended not to drill completely through the bone; leave a thin layer intact to avoid contacting the dura with the drill bit, as the bit can penetrate it. Exercise particular caution when drilling over the sagittal sinus; do not drill entirely through the bone in this area to prevent vascular injury to the sinus.

**Figure 9:**
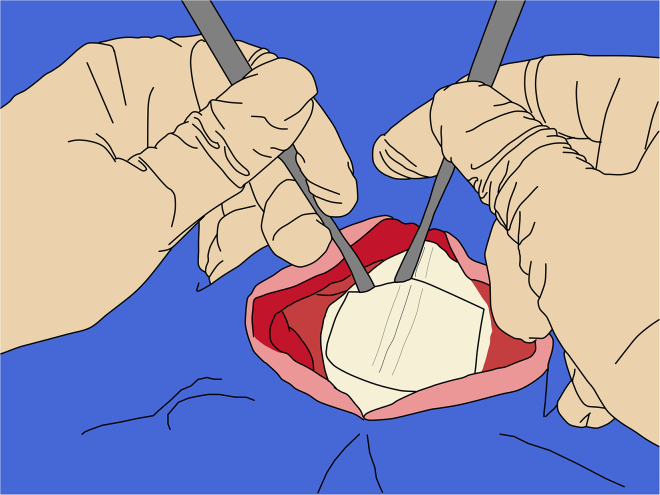
Removal of the bone flap
  d. Use a periosteal elevator to gently press against the underside of the bone flap and separate any adhesions without damaging the dura.
  e. Continue lifting the flap while progressively detaching it.
    **CRITICAL:** Avoid inverting or pushing down the opposite edge of the flap, as this may place pressure on the underlying cortex.
  f. Store the bone flap in a sterile bowl filled with sterile saline solution. It is common to observe minor bleeding after removing the bone.
  g. Irrigate the dura surface, and if the bleeding is excessive or does not stop after a few minutes, apply a small piece of surgical neurosurgical patty to achieve hemostasis.
  h. Cut the patty to the size of the bleeding spot to avoid excessive weight on the brain during surgery.
    **CRITICAL:** Remove the patty with irrigation before closure. In case of significant hemorrhage, use gel-foam to minimize the risk of intracranial bleeding after the surgery.
  i. To replace the bone flap after the injections, suture lines can be used to secure it.
  j. Removing any dural adhesions around the craniotomy margin using a periosteal elevator.
  k. Gently press the elevator towards the inner surface of the bone and remove the adhesions.
  l. To replace the bone flap after the injections, suture lines can be used.
  m. Using a bore drill bit, create suture holes approximately 3 mm from the edge of the craniotomy.
    **CRITICAL:** Angle the drill slightly to facilitate easier suture placement.
  n. While drilling, place a brain spatula underneath the bone and press firmly against the bone to shield the dura.
    ***Note:*** The frontal bone is generally not suitable for suture placement. In these surgeries, it is preferable to place two holes on the lateral sides and two on the caudal edge of the craniotomy. Drill corresponding suture holes on the bone flap as well.
  o. Use rongeurs to smooth any sharp edges along the bone flap and the craniotomy. In cases of bone bleeding, apply bone wax to achieve hemostasis.
    **CRITICAL:** Be sure to remove the excess bone wax before opening the dura to prevent wax fragments from entering the surgical field or intracranial space, as bone wax is typically non-absorbable.
34. Measuring the sinus:
  **CRITICAL:** Measure the sagittal sinus as soon as possible after bone flap removal, as delays can significantly affect targeting accuracy.
  a. Mount the micromanipulator with the injection circuits onto the stereotaxic bars.
    ***Note:*** If disposable drapes are used, carefully cut through them with a sterile blade to expose the arms.
    **CRITICAL:** Take special care to avoid bending the injection needle.
  b. Position the micromanipulator at the most caudal target along the AP axis.
    ***Note:*** If using a micromanipulator with fine AP adjustments, it is recommended to set the coarse AP position on the first (most caudal) target with the fine adjustment dial set to zero. Once the coarse position is fixed, the fine AP adjustment can be used to step through each target location.
  c. After setting the AP position, adjust the ML and DV until the tip of the needle gently touches the middle of the sagittal sinus (Figure 10).
    **CRITICAL:** Take special caution not to compromise the sinus.

**Figure 10:**
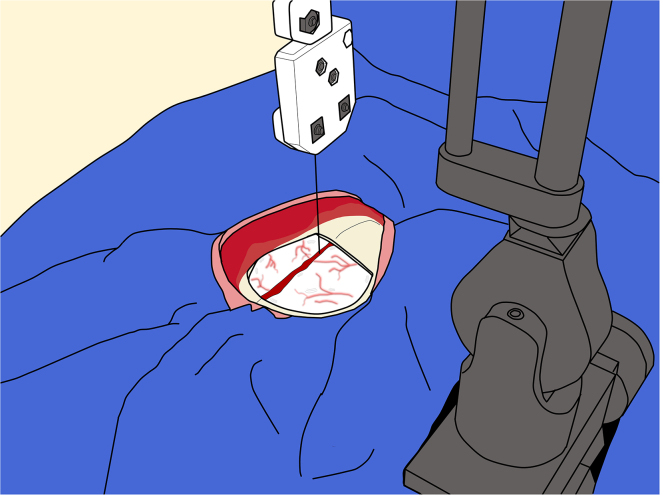
Measurement of the sagittal sinus
  d. Record the ML and DV on the manipulator and repeat this procedure for each target along the AP axis.
    **CRITICAL:** All coordinates must be independently cross-checked by a second person throughout the procedure to minimize the risk of human error.
35. Dura opening:
  ***Note:*** Opening the dura mater helps maintain needle alignment, since the dura is a relatively tough tissue that can bend or deflect the needle’s trajectory. Maintain at least 5 mm margin between the dura incision and the edges of the craniotomy.
  a. Using a #11 blade on a #7 handle, make a small initial nick in the dura, keeping the back of the blade parallel to the cortical surface to avoid injury to the underlying brain tissue (Figure 11).

**Figure 11:**
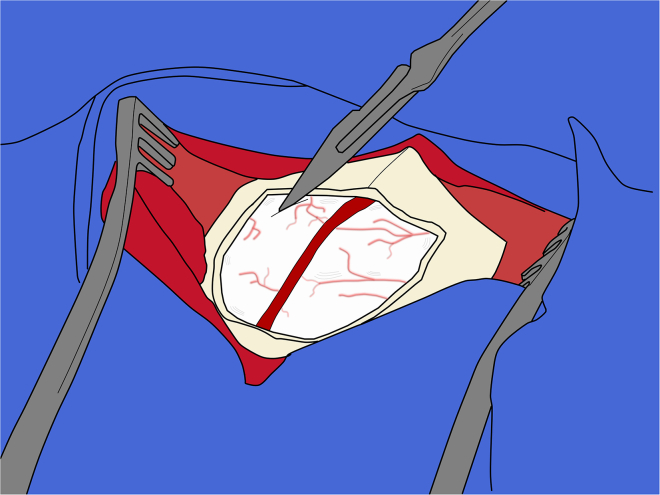
Initial incision of the dura mater
  b. Then, using fine tissue forceps, gently grasp and lift the dura at the nicked edge.
  c. Using the #11 blade or tip of iris scissors, carefully penetrate the dura, keeping the tips far from the cortex (Figure 12).

**Figure 12:**
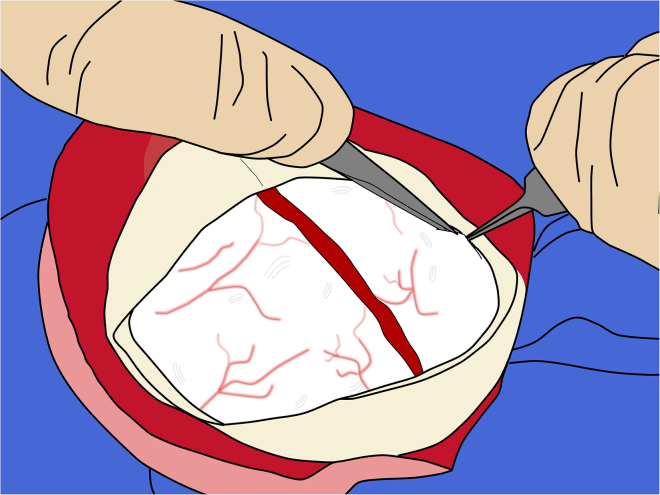
Opening of the dura mater
  d. Once you see cerebrospinal fluid, continue the incision using the scissors.
    **CRITICAL:** Keep the blades slightly open (1 to 2 mm) and glide them along the dura rather than using a cutting or closing motion (Figure 12). At the rostral and caudal ends of the incision, take extra care to avoid injuring the sagittal sinus.
36. Viral injection:
  a. Verify flow through the injection circuit by initiating virus efflux.
    **CRITICAL:** Begin with a relatively high flow rate (e.g., 20 μL/min) to first fill up the dead volume of the circuit.
  b. Once the circuit is primed, reduce the rate to 1 to 5 μL/min and continue until viral efflux is visually confirmed at the needle tip.
  c. Then, stop the flow or reduce it to a very low rate (e.g., 0.01 μL/min).
  d. Adjusting AP and ML positions for the injection target, lower the needle and stop just above the cortical surface, and inspect the anticipated penetration site for any large blood vessels in the pia mater.
    **CRITICAL:** If a vessel is present, minimally adjust the AP or ML to avoid it. If repeated minor adjustments fail to identify a vessel-free entry point, skip that injection site and proceed to the next planned target. Once clear, gently penetrate the cortex and advance the needle to the target depth. Note that the pia mater may resist penetration, particularly when using needles with shallow bevel angles (e.g. 45°). Optionally, a minimal flow rate (e.g. 0.01 μL/min) can be maintained during the descent to help prevent needle blockage by tissue (Figure 13).

**Figure 13:**
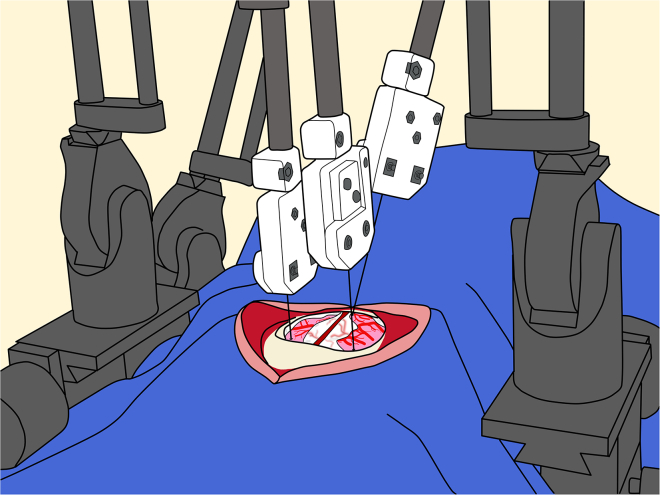
Viral injection
  e. After reaching the desired DV coordinate, set the injection pump to the intended rate.
    **CRITICAL:** To minimize tissue damage during injection, it is important to irrigate the dura and exposed cortex with sterile saline at least every 2**–**3 min. Redirecting the surgical light can also help prevent overheating and drying the tissue.
  f. Following the completion of the injection, wait for 10 min before withdrawing the needle to minimize the risk of backflow of the injected solution due to positive pressure at the injection site.
  g. Retract the needle very slowly over the first 2**–**3 mm, to avoid creating negative pressure that could draw virus along the penetration trajectory.
  h. Confirm viral flow at the end of each injection once the needle is out of the brain.
    **CRITICAL:** For multiple injections along a penetration track within a single target structure, it is recommended to proceed from the most ventral site to the more superficial one. In such cases, the waiting period between injections may be reduced to 1 min, as backflow is not a major concern.
37. Closing:
  a. Before closure, remove the micromanipulators from the stereotaxic arms and flush the cortical surface with normal saline, ensuring that no blood clots and no bleeding remain.
38. Closing the dura:
  a. Using fine tissue forceps and Castroviejo needle holder, suture the dura with either absorbable or nonabsorbable monofilament sutures (size 5-0 or 6-0) on a tapered needle (Figure 14).

**Figure 14:**
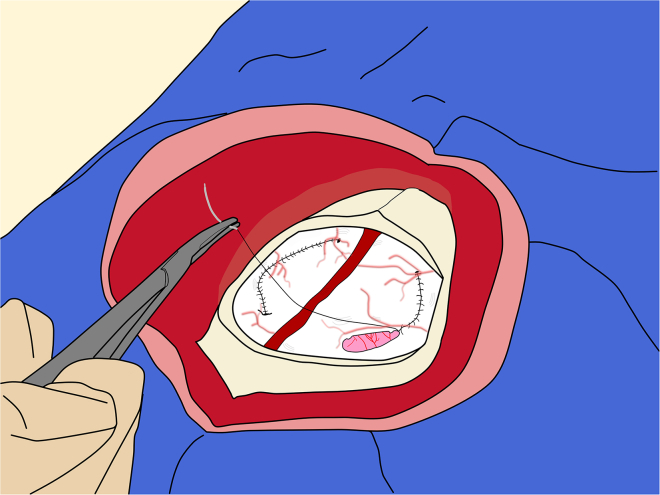
Closure of the dura mater

***Note:*** A simple continuous suture pattern is preferred, as it minimizes the amount of suture material left on the dura. Sutures should be placed approximately 1 to 1.5 mm apart. The dura is elastic, and in many cases, it is still possible to achieve primary closure despite initial appearance. When attempting dural closure, take small bites (approximately than 1 mm from the margin) to ensure that the full closure is possible.

**CRITICAL:** In case of extreme brain swelling, avoid placing tension on the tissue by attempting to close the dura. In such situations, it is best to leave the dura open and instead cover the area with an artificial dural graft.

39. Bone flap replacement:
  ***Note:*** Use a multifilament absorbable suture (size 3-0 or 2-0) for each suture hole.
  a. Begin by threading the suture tails through the holes on the craniotomy margins.
  b. Use fine forceps to pull the sutures through, if necessary.
  c. Pass the sutures through the corresponding holes on the bone flap.
    **CRITICAL:** Ensure the suture lines are not entangled before proceeding.
  d. Tie each suture using a square knot, formed by placing two single knots in opposite directions.
  e. Gradually tighten the sutures in multiple steps, alternating sides to prevent uneven tension that could misplace the bone flap.
    **CRITICAL:** Avoid over-pulling the sutures so as not to break the suture lines.
  f. Once the bone flap is properly aligned and stable, secure each suture with one or two additional single knots (Figure 15).

**Figure 15:**
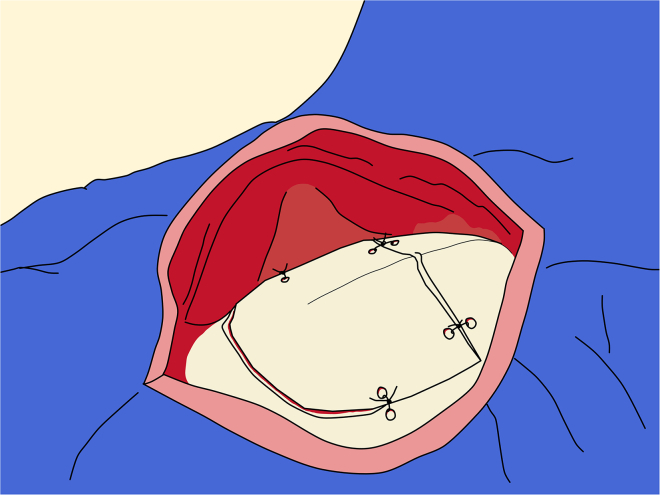
Replacement of the bone flap
40. Closing fascia:
  a. Use multifilament absorbable suture lines (size 3-0) to close the galea in a simple continuous pattern.

**CRITICAL:** Sutures should be spaced approximately 3 to 4 mm apart (Figure 15). Ensure that both the rostral and caudal ends of the fascia are securely closed.

41. Closing skin:
  a. Use a monofilament absorbable suture line (size 3-0) in an intradermal pattern to close the skin.

***Note:*** Alternatively, a monofilament non-absorbable suture line (size 3-0 or 2-0) can be used in a horizontal mattress pattern, placing approximately 2 mm from the skin margin and 4 mm apart. While the horizontal mattress pattern often overly everts the incision margins, the advantage is that the suture lines remain on either side of the incision rather than crossing it, reducing the likelihood that the animal can pick at the sutures. Continuous patterns leave less foreign material in the wound than interrupted patterns, thereby reducing postoperative irritation and improving healing. However, continuous sutures can be more prone to complications if they become loose, for example, due to animal interference or infection. Nonabsorbable sutures can be removed 10**–**14 days after the surgery.

42. Recovery:
  a. Following extubation, place the animal in an Intensive Care Unit (ICU) with heat and supplemental oxygen, and monitor continuously until signs of consciousness are observed.
  b. Once fully conscious, provide an oral fluid rehydration drink (e.g., Prang). On the following day, offer moistened chow and fruit, with free access to water and the oral rehydration drink.
  c. Monitor the animal at least twice daily during the postoperative period for neurological or other clinical signs.
  d. Assess mentation (normal, obtunded, stuporous, comatose), mobility and coordination, signs of seizures (ataxia, tremors, excessive salivation, loss of consciousness), and other neurological abnormalities such as head tilt, head pressing, or star-gazing.
  e. Because brain swelling may peak 48**–**72 h after surgery, maintain animals in the ICU for a minimum of 3 days postoperatively.

### Post-operative medications and monitoring

**Timing: 2 weeks**

This section describes post-operative care, including continued administration of medications and daily monitoring to assess recovery and detect potential complications.

43. See supportive medications section for post-operative medications and monitoring.

***Note:*** See troubleshooting 2 if a seroma occurs.

### Terminal brain extraction for *ex vivo* slice electrophysiology

**Timing: 3 h**

This section describes terminal brain extraction optimized for ex vivo slice electrophysiology, including pre-perfusion surgical preparation, transcardial perfusion, rapid brain removal, and tissue processing. These steps are designed to preserve tissue viability and physiological integrity for high-quality electrophysiological recordings.

***Note:*** The brain may be removed 5**–**8 weeks after the injection surgery, which represents a commonly used window that yields reliable transgene expression for electrophysiological recordings. The total elapsed time from the start of perfusion to brain removal and gross blocking should be kept to approximately 25 min. If viable tissue is not required (e.g., for histological analyses), a standard transcardial perfusion with formaldehyde may be performed following the same perfusion and brain extraction steps, and step 44 (surgical opening of the skull) can be omitted.

**Figure 16:**
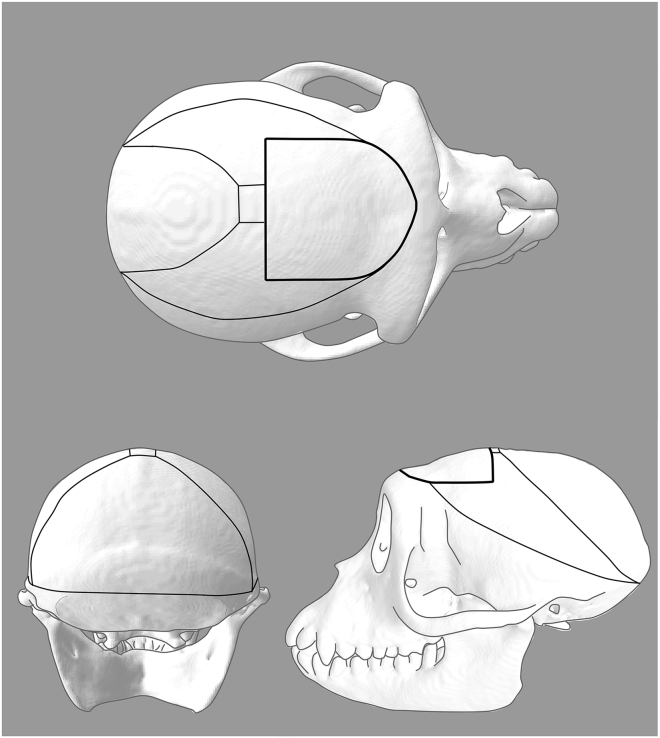
Skull drilling pattern Thick lines denote the craniotomy drilling pattern for injection surgery; thin lines denote the drilling pattern used for terminal brain extraction.
  e. Using two periosteal elevators, gradually lift the bone flaps without fully removing them.
    **CRITICAL:** Proceed cautiously, as the original bone flap may be adherent to the dura and other meningeal layers.
    ***Note:*** See troubleshooting 3 if you encounter a meningeal adhesion during the surgical opening.
45. Perfusion:
  a. Transfer the animal to a necropsy setup, in a supine position with the head and exposed skull resting on saline-soaked gauze.
  b. Using a #22 blade, make a midline incision between the clavicles extending caudally to the lower pelvic region.
  c. Perform thoracotomy, using blunt scissors, then open the diaphragm and pericardium to expose the heart.
  d. Clamp the descending aorta in the lower thorax to ensure adequate perfusion of the brain.
  e. Using Mayo scissors, cut the right auricle to open the right atrium, allowing blood to drain from the cardiovascular system and lower blood pressure.
  f. Excise the apex of the heart to open the left ventricle.
  g. Insert a large-bore cannula with an olive tip through the apex tip into the left ventricle and advance into the ascending aorta.
  h. Secure the cannula by clamping the apex to prevent leakage.
  i. Using a peristaltic pump at a flow rate of 100 ml per minute connected to a reservoir with 1.5–2 L of ice-cold fully oxygenated and consciously bubbled ssACSF, perfuse the animal until the blood vessels are clear (approximately 10**–**20 min depending on the size of the animal).

***Note:*** Perfusion is assisted by residual cardiac function; the heart should continue beating throughout the procedure.

**Figure 17:**
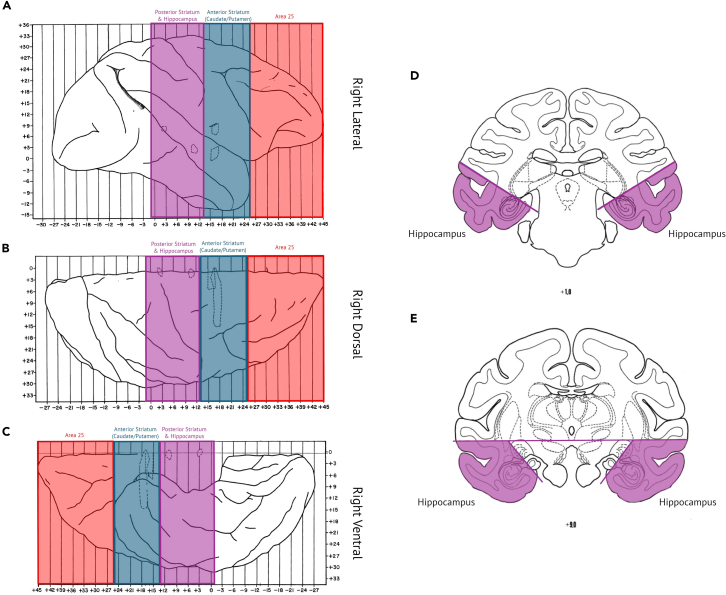
Brain blocking guide (A–C) Lateral, dorsal, and ventral views of the right hemisphere showing the approximate positions of coronal cuts used to generate tissue slabs. Shaded regions mark the anatomical divisions used as a reference during blocking: posterior striatum and hippocampus (purple), anterior striatum, including the caudate and putamen (blue), and anterior cortical regions, including area 25 (red). (D and E) Coronal sections illustrating representative cuts used to separate the hippocampus from surrounding structures.
  c. Block the brain in the coronal plane, starting with the most rostral cut (e.g., temporal pole).
  d. Remove the coronal slabs from the mold and hemisect to separate the hemispheres.
  e. Further block individual structures from each coronal slab as needed (see Figure 17).
  f. Immediately transfer tissue blocks into sealed containers with cold oxygenated ssACSF for storage or transport to host labs for acute ex vivo brain slice preparation for electrophysiological recording.

***Note:*** There is no required minimum storage duration prior to slicing, and blocks may be processed immediately if a vibratome is available. In our experience, storage delays of approximately 15**–**30 min under cold, oxygenated conditions preserve tissue suitable for electrophysiological recordings.

44. Surgical opening of the skull:
  ***Note:*** To facilitate extraction and minimize the post-mortem interval, the skull and soft tissue should be prepared under anesthesia prior to perfusion. Anesthesia should be continuously maintained throughout the perfusion procedure until completion of exsanguination.
  a. Follow the steps described earlier for preparation, draping, and surgical opening.
    **CRITICAL:** Take special care when encountering adhesions formed after the initial injection surgery. Avoid applying excessive force, as the bone flap may not be fully integrated and unnecessary pressure can damage the underlying brain tissue.
  b. Detach the temporalis muscles from their bony origins on the bone and expose the occipital ridge to the level of the zygomatic arch.
  c. Detach the trapezius muscle from the occipital ridge to expose its ventral aspect.
  d. Drill the skull following the pattern shown in Figure 16.

46. Brain extraction:
  ***Note:*** Following transcardial perfusion with chilled sucrose-substituted artificial cerebrospinal fluid (ssACSF), the brain should be removed as rapidly as possible. The total elapsed time from the start of perfusion to brain removal and gross blocking should be kept to approximately 25 min.
  a. Turn over the animal to a prone position and remove the drilled bone flaps using rongeurs. Ensure complete removal of the remaining occipital bone.
  b. Use forceps and curved scissors, dissect the most posterior extent of the dura mater over the occipital lobes, then along the midsagittal plane to remove the falx cerebri as well as the tentorium cerebelli.
  c. Transect the cervical spinal cord and bluntly dissect the cranial nerves using the flat end of the scalpel handle.
  d. Remove the brain from the skull in a caudal to rostral direction, carefully dissecting any remaining attachments.
47. Blocking:
  a. Immediately place the extracted brain into a brain mold and irrigate continuously with cold oxygenated ssACSF.
  b. Use the mold to cut full coronal plane tissue slabs. Figure 17 illustrates the coronal incision planes for the structure presented in this paper.

48. Slicing:
  a. Trim gross brain blocks containing the structure of interest (approximately 1–1.5 cm or smaller).
  b. Adhere the trimmed tissue block to a specimen stage using cyanoacryate (e.g., crazy glue).
  c. Section the tissue into 250**–**400 μm slices using a vibratom (e.g., Leica Microsystems VT-1200S), while submerged in ice-cols oxygenated ssACSF.
  d. Transfer individual slices are to a submerged incubation chamber containing oxygenated warmed (32°C**–**34°C) ssACSF.
  e. Incubate slices for 30 min and maintained at at 32°C**–**34°C.
  f. After incubation, maintain slices under sterile conditions with stable osmolarity for several days if long term storage is required.
  g. Inspect the incubation solution at least once daily for contamination or evaporation.
  h. Replace the solution as needed to maintain sterility and osmolarity.
    ***Note:*** Slices can typically be maintained for 3**–**5 days without solution exchange if sterility and osmolarity remain stable. The frequency of solution exchange depends on chamber cleanliness, evaporation rate, and environmental conditions.
  i. Allow slices to recover for at least one hour after slicing.
  j. Transfer individual slices to the recording setup for electrophysiological experiments.

## Expected outcomes

**Figure 18:**
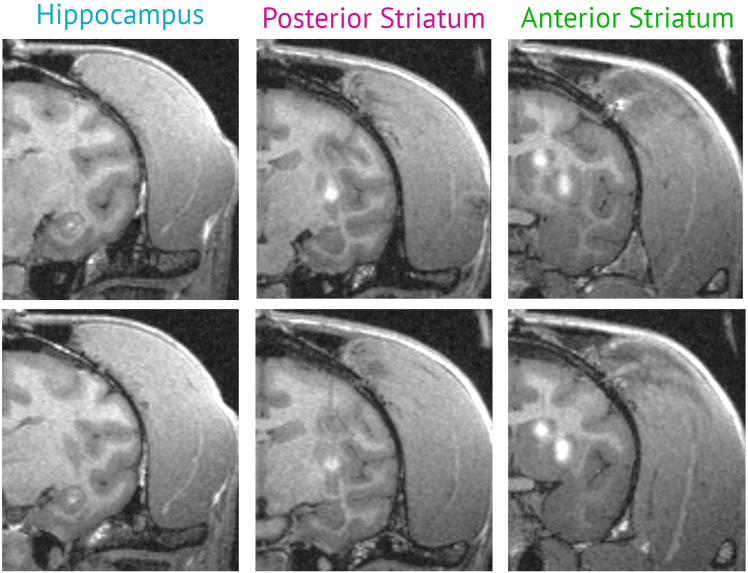
Post-operative MRI scan Regions of hyperintense signal indicate the presence of manganese added to the viral solution, confirming successful liquid delivery and validating targeting accuracy. Note that these injection parameters are not intended to uniformly cover the entire volume of the targeted structures; however, they reliably produce sufficient levels of transfection for the studies cited in this paper.

It is possible to assess injection performance using manganese solutions^12^ in a post-operative MRI scan. Figure 18 illustrates a coronal slice from post-operative scans performed immediately after the surgical procedure, showing microinjection targeting presented in Table 1. In these scans, successful liquid deliveries typically appear as localized regions of contrast enhancement at the intended target sites, showing hyperintensity on T1-weighted images. Following intracranial delivery of enhancer-driven adeno-associated viruses (AAVs) and an expression period of 6**–**8 weeks, virally labeled neurons can be identified by robust reporter expression and appropriate co-localization with molecular markers assessed by immunohistochemistry. In addition, infected neurons exhibit electrophysiological properties consistent with their expected cell types when examined in acute brain slices.

## Limitations

The main limitation of stereotaxic injection procedures arises from the reliability and accuracy of the imaging data. A voxel size of approximately 0.5 mm is commonly used in nonhuman primate brain MRI scans. In this protocol, multiple scans are routinely acquired within a single session and averaged to improve spatial accuracy. The primary limitation of the injection circuit introduced in this paper is its poor performance with substances that degrade or lose stability during loading or slow delivery through the tubing and connectors. However, in our experience, it has been reliable for virus injections.

## Troubleshooting

### Problem 1

Clogged injection circuit (step 4).

On rare occasions, the injection circuits may become clogged, either due to small fragments in the viral solution or mineral oil, or because tissue is pushed into the needle during brain penetration when targeting multiple sites within the same structure.

### Potential solution

• Alternating flushes of sterile water and 70% ethanol can often clear a clogged circuit, especially when using a pump to maintain steady positive pressure and injection flow. If clogging occurs during surgery following a tissue penetration, it is likely caused by tissue obstructing the needle tip. In this case, use a pair of fine forceps to gently wipe the tip of the needle and remove debris. Afterwards, verify that the injection flow is restored.

### Problem 2

Seroma fluid accumulation (step 43).

Accumulation of seroma is relatively common after such extensive surgeries involving dissection of multiple tissue layers, detachment of the temporalis muscles, and large craniotomies. This is due to a large area of dead space and is more commonly seen in larger animals (e.g., males). Excessive seroma buildup typically becomes apparent in the second postoperative week.

### Potential solution

• Pay close attention to the wound closure. Ensure that the bone flap is properly repositioned, so its margins are well aligned with the surrounding skull and securely tighten the suture lines connecting the flap to the bone.
• To minimize dead space, suture the posterior portions of the temporalis muscles together when anatomically possible (note that there are variations and some animals, particularly females, may have less medial overlap).
• Close the galea as tightly as possible to prevent fluid accumulation beneath it.
• Maintain strict sterility throughout the surgery, keep the tissues moist, and irrigate generously before closing each layer. In cases of excessive seroma formation, consider draining the fluid in consultation with a veterinarian. The decision to drain is based on two primary factors:
  ○ Evidence of impaired wound healing, including inflammation, irritation, suture dehiscence, or the animal manipulating the surgical site; and
  ○ Progressive enlargement of the seroma causing increased skin tension, often producing a conical appearance over the cranium. Even in the absence of clear signs of inflammation, a conical appearance indicates rising tension that may compromise incision healing. It is recommended to culture fluid drained from the surgical site to evaluate for developing infection.
• Additionally, a diuretic, such as furosemide (1 mg/kg) once daily, can be initiated to decrease fluid accumulation in consultation with a veterinarian.

### Problem 3

Meningeal adhesion (step 44).

During the healing process, the dura and pia mater can fuse together. Infection can promote adhesion formation. Moreover, mechanical trauma from needle penetrations may lead to localized meningeal adhesions. This issue is particularly important for dorsal cortical targets, as removing such adhesions during brain extraction can risk cortical damage.

### Potential solution

• To reduce the likelihood of meningeal adhesion, minimize tissue trauma during needle entry and thoroughly irrigate the surgical site to remove any blood clots before dura closure. As described in this protocol, the use of 30-gauge needles with a 45 degrees lancet-style bevel further helps minimize tissue trauma during penetration.
• More importantly, avoid overlapping the dura incisions with penetration sites. Before opening, you can estimate the target location by a sterile tiller on the skull by opening the bone flap about 10 mm from the target sites and keep the dural opening a few millimeters far from bone margin.

## Resource availability

### Lead contact

Further information and requests for resources and reagents should be directed to and will be fulfilled by the lead contact, Reza Azadi (reza.azadi@nih.gov).

### Technical contact

Technical questions on executing this protocol should be directed to and will be answered by the technical contact, Reza Azadi (reza.azadi@nih.gov).

### Materials availability

This study did not generate new unique reagents.

### Data and code availability

• All data reported in this paper will be shared by the lead contact upon request.
• All code used in this paper will be shared by the lead contact upon request.
• Any additional information required to reanalyze the data reported in this paper is available from the lead contact upon request.

## Acknowledgments

We thank the Section on Instrumentation (NIMH) for their critical support. Anatomical MRI scanning was carried out in the Neurophysiology Imaging Facility Core (NIMH, NINDS, NEI). This research was supported by the Intramural Research Program of the National Institutes of Health (NIH) ZIA MH002928 (to B.A.). This research was supported by the Intramural Research Program of the National Institutes of Health (NIH). The contributions of the NIH authors are considered Works of the United States Government. The findings and conclusions presented in this paper are those of the authors and do not necessarily reflect the views of the NIH or the U.S. Department of Health and Human Services.

## Author contributions

A.C.T. and P.H. prepared the illustrations and original manuscript with guidance from R.A.; A.C.T., P.H., R.A., A.S.P., A.M., and M.A.G.E. developed and performed the surgical procedure; A.C.C., K.A.P., C.J.M., and M.A.G.E. developed and performed the terminal brain extraction; K.C. and C.R. helped develop the injection circuit; K.A.G. performed and supervised the anesthesia and medical procedures; B.B.A. supervised the overall project; and all authors reviewed the manuscript.

## Declaration of interests

The authors declare no competing interests.

## Supplemental information

Methods S1. Injection circuit holder, related to Step 1

Methods S2. Stereotaxic targeting calculator, related to Step 7
