# Supplementary material for: Surgical protocol for precise and high-throughput viral injections in rhesus monkey brain
Source: STAR Protoc. 2026 Apr 28;7(2):104522. doi: 10.1016/j.xpro.2026.104522 (PMC13136701; doi:10.1016/j.xpro.2026.104522)
Supplement: Methods S2. Stereotaxic targeting calculator, related to Step 7 [file mmc2.zip › Stereotaxic Targeting Calculator Note.docx]

## **Supplementary Excel File: Stereotaxic Targeting Calculator**

This Excel file is provided to assist with calculating stereotaxic injection coordinates based on pre-operative MRI measurements and intraoperative landmarks.

###

### **Sheet: “Zero”**

- Enter the **ear-bar coordinates measured from the MRI scan** in cells **D4–D6** (left side) and **F4–F6** (right side).
- Enter the **ear-bar coordinates measured on the stereotaxic frame** in cells **D9–D11** (left side) and **F9–F11** (right side).
  - When using **Kopf micromanipulators**, it is convenient to keep the AP micro set to **25 mm on the left** and **0 mm on the right**. This configuration allows injections to proceed from the most caudal target to the most rostral.
- **AP macro offsets** (cells **D13** and **F13**) are automatically calculated based on the most caudal targets entered in the **“Left hemisphere”** and **“Right hemisphere”** sheets.
  - This design keeps the AP macros aligned with the most caudal targets, allowing the full **25 mm AP micro range** to be used during surgery and minimizing the need to adjust the AP macro intraoperatively.
- **Final AP macro positions** (cells **D15** and **F15**) indicate the AP macro values to be set on the micromanipulators.
  - These values depend on entries in the **“Left hemisphere”** and **“Right hemisphere”** sheets, which must be completed before using this section.

**Sheets: “Left hemisphere” and “Right hemisphere”**

- Enter target coordinates derived from the MRI in the **“From scan target”** columns (**O, P, Q**).
- For each target, enter the corresponding **sagittal sinus coordinates** from the same coronal slice into the **“From scan sagittal sinus”** columns (**S, T, U**).
  - Example values provided in the file are based on targeting performed on the **macaque template NMT v2**.
- During surgery, measure the sagittal sinus position at each AP level and enter the values into columns **L and M**.
- Use columns **C, D, and E** to obtain the final stereotaxic coordinates for targeting and injection.
- Columns **G, H, and I** can be used to record injection progress and notes during the procedure.
